# SPABBATS: A pathway-discovery method based on Boolean satisfiability that facilitates the characterization of suppressor mutants

**DOI:** 10.1186/1752-0509-5-5

**Published:** 2011-01-11

**Authors:** Lope A Flórez, Katrin Gunka, Rafael Polanía, Stefan Tholen, Jörg Stülke

**Affiliations:** 1 Department of General Microbiology, Georg-August-University of Göttingen, Göttingen, Germany; 2 Department of Clinical Neurophysiology, Georg-August-University of Göttingen, Göttingen, Germany

## Abstract

**Background:**

Several computational methods exist to suggest rational genetic interventions that improve the productivity of industrial strains. Nonetheless, these methods are less effective to predict possible genetic responses of the strain after the intervention. This problem requires a better understanding of potential alternative metabolic and regulatory pathways able to counteract the targeted intervention.

**Results:**

Here we present SPABBATS, an algorithm based on Boolean satisfiability (SAT) that computes alternative metabolic pathways between input and output species in a reconstructed network. The pathways can be constructed iteratively in order of increasing complexity. SPABBATS allows the accumulation of intermediates in the pathways, which permits discovering pathways missed by most traditional pathway analysis methods. In addition, we provide a proof of concept experiment for the validity of the algorithm. We deleted the genes for the glutamate dehydrogenases of the Gram-positive bacterium *Bacillus subtilis *and isolated suppressor mutant strains able to grow on glutamate as single carbon source. Our SAT approach proposed candidate alternative pathways which were decisive to pinpoint the exact mutation of the suppressor strain.

**Conclusions:**

SPABBATS is the first application of SAT techniques to metabolic problems. It is particularly useful for the characterization of metabolic suppressor mutants and can be used in a synthetic biology setting to design new pathways with specific input-output requirements.

## Background

A holistic understanding of cellular metabolism is central to systems biology and metabolic engineering: In order to amplify the flux through production pathways in industrial strains we have to understand how the metabolic network responds to our interventions.

Several methods can suggest rational interventions that may lead to favourable industrial phenotypes (see [1] for a review). Their goal is to optimize the distribution of metabolic fluxes towards the product of interest, either directly (e.g. FBA, MOMA or ROOM) or indirectly by coupling it to another characteristic (e.g. OptKnock) that facilitates further strain improvements via mutation and screening.

While these methods can predict a final flux distribution, they do not predict the range of genetic and metabolic responses of the organism after the targeted mutation. At the same time, it would be highly desirable to have tools that may predict these responses, since they can suggest ways to generate more stable strains, or accelerate the adaptation to an intended optimal flux. The challenge of the question is the need to understand why particular genetic responses make sense in an evolutionary setting. Thus, the ultimate question is: Which parallel pathways - that were not active previously - result in an adaptive advantage under the screening conditions?

Pathway analysis has received increased attention due to the reconstruction of genome scale metabolic networks for many organisms. These methods can be divided into two categories: stoichiometric and path oriented (see [2] for a review). The first approach generates all pathways that conform to the pseudo-steady-state assumption for internal metabolites. However, it presents two problems: the number of predicted pathways is in the order of millions for genome scale models, making the approach totally intractable for the question at hand [3]. Its second shortcoming is the constraint imposed by the pseudo-steady-state assumption for internal metabolites. This assumption may rule out feasible pathways or (in case we include a large number of "freely available" metabolites) result again in a combinatorial explosion of pathways. The alternative approach - path oriented pathway reconstructions - is advantageous since it usually generates a small (and thus tractable) set of possible pathways. This is due to the choice of starting and ending metabolites and heuristics on the characteristics of the "optimal" pathway. However, the path-oriented approach may result in unrealistic pathways that consume internal metabolites not present in sufficient quantities inside the cell.

What is needed is an algorithm that reconstructs stoichiometrically balanced pathways in increasing order of complexity, with relaxed mass-balance constraints in comparison to the traditional pseudo-steady-state restriction.

A solution based on mixed-integer linear programming (MILP) has been suggested by de Figueiredo *et al. *[4], but it has not been used in an evolutionary context so far. Here we describe the use of Boolean satisfiability (SAT, [5]) for the reconstruction of alternative pathways in metabolic networks. Given a set of basal metabolites (that are considered freely available) and a set of target metabolites (whose concentration *must *increase), our SAT method constructs the shortest pathway between the basal and target sets (SPABBATS) of metabolites that is stoichiometrically balanced, while allowing the concentration of the intermediate metabolites to increase, if needed. The constraints are more relaxed than the ones for e.g. flux balance analysis, thus retaining the metabolically significant pathways. Using the algorithm iteratively, we obtain a prioritized list of pathways, whose elements can be tested individually by common molecular biology techniques.

To demonstrate the power of this concept, we applied the SPABBATS algorithm to a complex physiological problem, which is a result of an evolutionary experiment. We have elucidated a novel pathway of glutamate degradation present in the metabolic network of *B. subtilis *that had been decryptified upon inactivation of the normal glutamate catabolic genes. By using our SAT approach, we proposed four different new pathways that could be present in the mutant to utilize glutamate as single carbon source. These predictions were experimentally tested and revealed that one of these pathways was indeed active in the mutant strain and that this novel "suppressor" pathway is required and sufficient for glutamate utilization. This proves that the results of our approach correspond to valid metabolic alternatives for living cells.

## Results

### Isolation of a mutation that allows a bypass of the glutamate dehydrogenase for the utilization of glutamate

Glutamate is the most abundant metabolite in a bacterial cell. Although its exact concentration in *B. subtilis *is unknown, it is known to account for about 40% of the internal metabolite pool of an *Escherichia coli *cell [6]. Glutamate serves as an osmotic regulator [7], as well as universal amino group donor in anabolism thus linking carbon and nitrogen metabolism [8]. In *B. subtilis*, at least 37 reactions make use of glutamate as cofactor for transamination [9].

**Figure 1:**
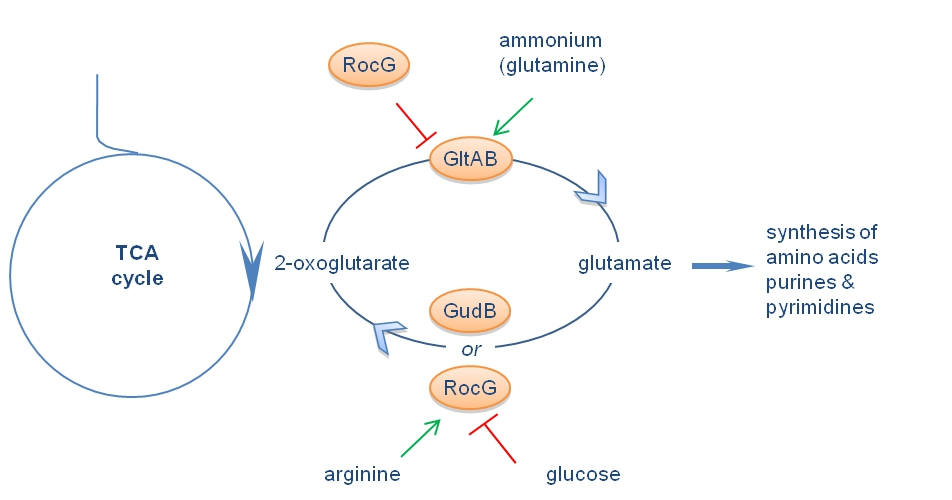
**Key reactions for glutamate biosynthesis and degradation in *Bacillus subtilis***. Glutamate is the universal amino group donor in all living cells and in that way links the carbon and nitrogen metabolisms. In *B. subtilis *the synthesis of glutamate depends on the glutamate synthase GltAB. In addition, the genome encodes two glutamate dehydrogenases, RocG and GudB, although the latter is inactive in the laboratory *B. subtilis *strain 168 (see text). The synthesis and degradation of glutamate are tightly regulated in response to the availability of carbon and nitrogen sources.

The key reactions of glutamate biosynthesis and degradation in *B. subtilis *are summarized in Figure 1. 2-oxoglutarate, an intermediate of the citric acid cycle, is aminated by the glutamate synthase, encoded by the *gltA *and *gltB *genes. Glutamate degradation to 2-oxoglutarate requires the glutamate dehydrogenase RocG. Additionally, the laboratory strain *B. subtilis *168 harbours a cryptic gene, *gudB*, coding for an inactive glutamate dehydrogenase. This gene is readily decryptified in *rocG *mutants [10,11]. In addition, RocG controls the expression of the *gltAB *operon and therefore prevents glutamate biosynthesis in the presence of arginine [12,13].

Inactivation of both the *rocG *and the *gudB *gene results in loss of any glutamate dehydrogenase activity and concomitant inability of the bacteria to utilize glutamate [10,11]. The *rocG gudB *double mutant strain GP28 grows poorly on SP medium (an amino acid-rich medium) due to the accumulation of degradation products of arginine metabolism [14]. However, cultivation of GP28 on SP plates eventually resulted in the isolation of a mutant (GP717) that carries a mutation inactivating the *gltB *gene, encoding a subunit of the glutamate synthase [11]. This mutation leads to glutamate auxotrophy and might therefore prevent the accumulation of intermediates of arginine degradation. We have observed that toxic intermediates of arginine degradation result in poor growth of mutants lacking a functional glutamate dehydrogenase (our unpublished results). If intrinsic glutamate synthesis is blocked by a mutation, such an accumulation of toxic intermediates might be reduced. A careful analysis of the mutant strain revealed that it had acquired the ability to utilize glutamate as the only source of carbon and energy. This might have resulted from a re-activation of the *rocG *or *gudB *genes or from the establishment of a novel pathway for glutamate utilization. We tested therefore the *rocG *and *gudB *alleles by PCR analysis. Both the transposon insertion in *rocG *and the replacement of the *gudB *gene by a chloramphenicol resistance gene were identical to the parent strain GP28. Clearly, a new pathway of glutamate degradation was activated in this suppressor mutant that was not active in the wild type and *rocG gudB *mutant cells.

### Development of a pathway-finding algorithm

The most reasonable hypothesis to explain the suppression was that the mutation had activated a redundant pathway that is inactive in the wild type strain in a medium with glutamate as single carbon source. Since glutamate is a highly abundant metabolite and is involved as a substrate in 20 reactions in *B. subtilis*, it was not obvious which mutation could have lead to glutamate utilization proficiency in *B. subtilis *GP717.

To address this problem by use of the power of bioinformatics, we developed an approach that harnesses the strengths of Boolean satisfiability (SAT) to find valid pathways (see Materials and Methods). It is able to find short pathways between a basis and a target set (SPABBATS) of metabolites that can operate in a sustained way. It is convenient for its focus on short pathways and the fact that it can calculate pathways that comply with the steady-state constraint. It also allows the relaxation of this constraint, by allowing some metabolites to accumulate if necessary.

**Figure 2:**
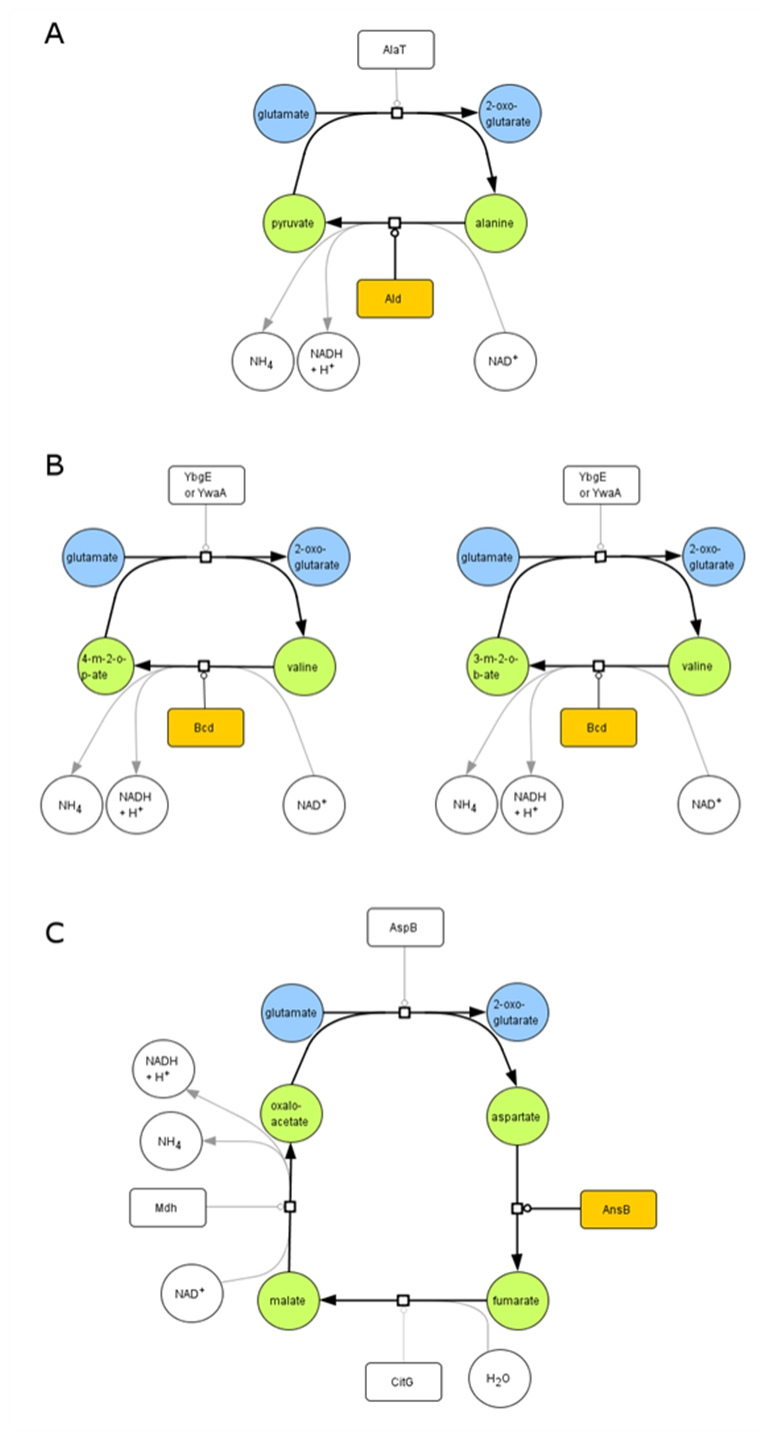
**Predictions of alternative pathways for glutamate utilization based on SAT techniques**. A *B. subtilis *strain (GP28) was constructed that lacks the glutamate dehydrogenases. An evolutionary adaptation resulted in a strain (GP717) that acquired the capacity to grow on glutamate as single carbon source. Using a SAT based search algorithm (see Materials and Methods) we predicted four alternative pathways that could be activate in the GP717. The genes coding for the enzymes in orange were analyzed further (see text). 4-m-2-o-p-ate = 4-methyl-2-oxo-pentanoate; 3-m-2-o-b-ate = 3-methyl-2-oxo-butanoate.

The first four pathways suggested by our algorithm are presented in Figure 2. In each case, the first step is a transamination reaction that leads to the production of 2-oxoglutarate. The substrate for transamination is then replenished via the remaining reaction(s) of the pathway. The first pathway (Figure 2A) involves transamination to form alanine and subsequent oxidative deamination of alanine by the alanine dehydrogenase Ald resulting in the net formation of 2-oxoglutarate. The next two pathways (Figure 2B) are very similar and involve enzymes of branched amino acid metabolism. In the transamination step, both pathways use the transaminases YbgE and YwaA. The branched chain amino acid dehydrogenase Bcd is then used for the oxidative deamination of the transamination products valine or leucine. Again, the net result of this pathway is the production of 2-oxoglutarate from glutamate. The last pathway (Figure 2C) requires four steps, (i) the reaction of the aspartate aminotransferase AspB, (ii) the deamination of asparate to fumarate by the aspartase AnsB, (iii) the fumarase reaction (CitG) of the citric acid cycle, and finally (iv) the oxidation of malate by the malate dehydrogenase Mdh. As described for the other pathways, this reaction sequence results in the net formation of 2-oxoglutarate from glutamate. Since the original mutant GP28 did not grow with glutamate as the single carbon source, it is obviously not able to use any of these proposed pathways suggesting that they were activated by a suppressor mutation in GP717.

### Experimental validation of the predictions

**Figure 3:**
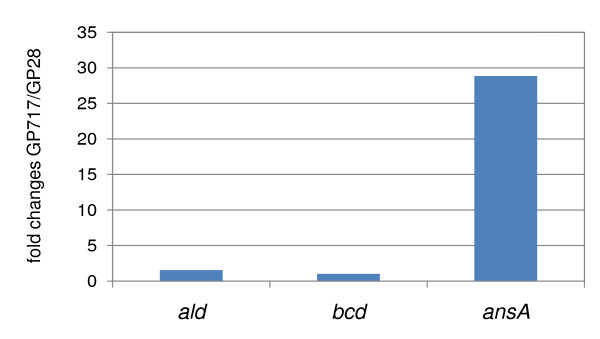
**Comparison of gene expression patterns between mutant and parental strains, based on the predictions of the SPABBATS algorithm for pathway analysis**. The predictions of the SPABBATS algorithm (see Figure 2) were further characterised by transcription analysis. The expression of the *ald *and *bcd *genes remains constant between the mutant (GP717) and parental (GP28) strains, suggesting that these genes are not involved in the newly activated catabolic pathway. In contrast, the expression of the *ansAB *operon is strongly increased in the mutant. This hints to a gain of function in the mutant strain that was analyzed further.

Our experiments were performed in minimal medium suggesting that the activity of transaminases was not limiting. Similarly, the two enzymes of the citric acid cycle (CitG and Mdh) are constitutively expressed [15-17]. Thus, the mutation may have affected the expression of one of the deaminases Ald, Bcd or AnsB. This hypothesis was tested by reverse transcription-real-time quantitative PCR. As shown in Figure 3, the levels of *ald *and *bcd *mRNA are comparable for the original mutant GP28 and the suppressor strain GP717. In contrast, a strong increase of the expression of the *ansAB *operon encoding the asparaginase and aspartase was observed for the suppressor mutant that was able to utilize glutamate. This observation suggests that it is the high-level expression of AnsB that allows glutamate utilization in GP717.

**Figure 4:**
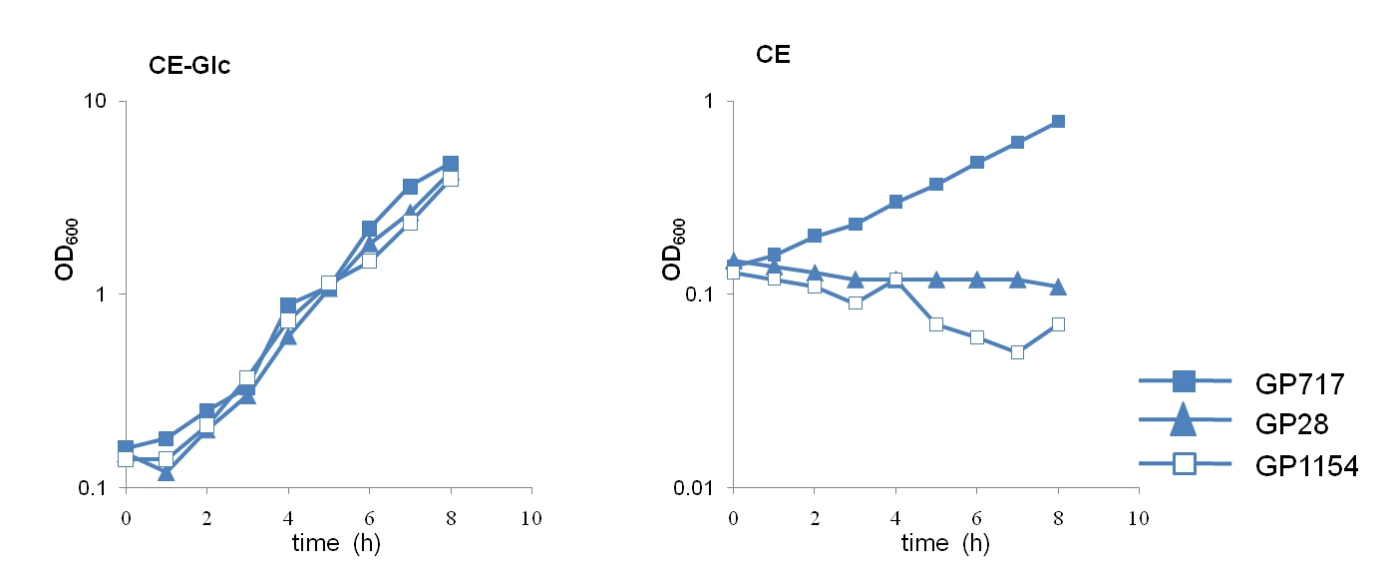
**Requirement of the aspartase gene in the alternative pathway for glutamate utilization**. The SPABBATS algorithm (see Figure 2) and the transcription analysis (see Figure 3) suggested that the overexpression of the asparaginase and aspartase genes (*ansAB*) is the cause for the metabolic gain of function of the mutant strain GP717. To prove this, the *ansAB *operon was deleted in the GP717 strain. The resulting strain GP1154 lost the capacity to utilize glutamate as single carbon source. This strongly indicates that the induction of the aspartase gene is required and sufficient for the newly activated catabolic pathway. CE = Minimal medium containing 0.5% glutamate, CE-Glc = CE medium with an addition 0.5% glucose.

The involvement of the aspartase AnsB in the novel glutamate utilization pathway was verified by analysing the effect of a deletion of the *ansAB *operon. Growth of the original strain GP28, the suppressor mutant GP717 and its isogenic Δ*ansAB *mutant derivative GP1154 in minimal medium with glutamate or with glutamate and glucose was recorded. As shown in Figure 4, all three strains were able to grow with glutamate and glucose. In contrast, the deletion of the *ansAB *operon reverted the capability of the suppressor strain of using glutamate as the single carbon source, and the Δ*ansAB *mutant GP1154 was unable to grow with glutamate as was the original strain GP28. This finding strongly supports the idea that the activity of the aspartase AnsB is the reason for the ability of the suppressor strain GP717 to utilize glutamate.

The *ansAB *operon is induced in the presence of asparagine due to inactivation of the AnsR repressor [18-20]. A comparative analysis of *ansAB *expression revealed about 30-fold induction by asparagine in GP28, whereas the expression levels were unaffected by the availability of asparagine in the suppressor mutant GP717 (data not shown). The observed induction in the wild type strain is good agreement with previous reports. The loss of regulation in GP717 and the high expression of the operon as compared to GP28 suggest constitutive *ansAB *expression that might be the result of an inactivation of the *ansR *repressor gene.

To test the hypothesis that inactivation of the AnsR repressor allowed glutamate utilization by GP717, we performed two tests: First, we deleted the *ansR *gene of the parental strain GP28 and tested the ability of the resulting strain GP811 to grow with glutamate as the single carbon source. Unlike GP28, this strain GP811 (Δ*ansR*) grew in CE minimal medium. Thus, inactivation of the *ansR *gene is sufficient to open a new pathway for glutamate catabolism. In a complementary approach, we complemented *B. subtilis *GP717 with a plasmid-borne copy of the *ansR *gene (present on pGP873) and tested the ability of the transformants to use glutamate. While the control strain (GP717 transformed with the empty vector pBQ200) grew well on CE medium, expression of AnsR from the plasmid completely blocked growth in this medium, *i. e. *the utilization of glutamate. This result confirms that a mutation in the *ansR *gene must be present in GP717 and that it is this mutation, which confers the bacteria with the ability to utilize glutamate via the new aspartase pathway.

To identify the mutation in *ansR*, we sequenced the *ansR *alleles of the parental strain GP28 and the glutamate-utilizing suppressor mutant GP717. While the wild type allele of *ansR *was present in GP28, a C-to-A substitution at position 107 of the *ansR *open reading frame was found in GP717. This mutation changes codon 36 from UCA (Ser) to UAA (stop) and results in premature translation termination and the formation of an incomplete and non-functional AnsR repressor protein.

Taken together, these experiments confirmed that the metabolic pathway predicted by the SPABBATS algorithm corresponds to a valid metabolic state of the *rocG gudB ansR *mutant strain GP717.

## Discussion

### Comparison of SPABBATS with other methods for metabolic analysis

Flux balance analysis [21] and the majority of methods derived from it are based on constraining the admissible intracellular flux space to steady-state and choosing an adequate optimality criterion to calculate intracellular fluxes. Commonly used optimization criteria are biomass production and the maximization of energy output.

Although these methods predict the essentiality of genes with high accuracy [9], they are less suited for the characterization of alternative metabolic pathways in viable mutants. On the one hand, by restricting the admissible intracellular flux to steady-state, they discard pathways where a by-product accumulates. Nonetheless, the cell is still viable if this by-product is consumed by other pathways in the cell, not directly related to the process that is studied. SPABBATS solves this problem by allowing a larger flux-space, where intermediate products can accumulate, if necessary.

On the other hand, the optimality criterion can be artificial. For instance, maximizing cellular growth might lead to a theoretical maximum growth rate, or a flux distribution that is as close to the wild-type flux as possible, but it is hard to argue that the regulatory network of the strain is directed to the same target. The pathways discovered by SPABBATS are a structural property of the network and do not depend on an extrinsic optimality criterion (beyond the number of reactions of the resulting pathway). For this reason, the resulting pathways can be interpreted objectively.

Other methods for structural decomposition (e.g. extreme pathways and elementary flux modes, see [2] for a review) rely on the same steady-state restriction of FBA related methods and for this reason share some of their disadvantages. Moreover, SPABBATS does not require the calculation of all possible pathways. Instead, it can be used iteratively to calculate pathways of increasing length, which results in a dramatic improvement in performance for finding relevant pathways in large networks.

An advantage over the method of de Figueiredo *et al*. [4] is that we do not make use of an optimization framework, but select for satisfiability instead. Similar problems in other areas of computational biology (e.g. [22]) show a performance improvement of SAT methods over traditional mixed-integer linear programming methods.

### Future perspectives

So far, our analysis of networks using SAT has been restricted to metabolic networks. Nonetheless, since SAT is especially suited for problems that involve Boolean constraints, it is possible to expand the analysis to regulatory networks. For *B. subtilis*, this implies the reconstruction of the metabolic network together with its regulatory complement. This reconstruction is in progress [23,24].

In parallel, we envision the development of novel SAT solvers that are optimized for the solution of metabolic constraints. This will result in the adoption of SAT based methods for metabolic engineering as well as for the design of synthetic circuits that are able to perform computations in the same way as their silicon-made counterparts [25].

## Conclusions

In this contribution we have shown the use of SAT techniques to discover alternative pathways that connect sets of starting and target species. In addition, we provided a proof of concept for the applicability of the algorithm. We started with a complex physiological problem in *B. subtilis*: the need to characterize a suppressor mutation that allowed growth on glutamate without glutamate dehydrogenases. SPABBATS predicted four potential pathways for glutamate utilization that were decisive to suggest target genes for experimentation. These experiments confirmed the validity of the SPABBATS' prediction, closing the cycle between modelling and wet lab experimentation.

SPABBATS relies on Boolean satisfiability (SAT) to construct the metabolic pathways. SAT has been used for the determination of haplotypes from sequenced genotypes [22], the analysis of genome biology networks [26], the understanding of myogenic differentiation [27], and the characterization of steady states of regulatory circuits [28,29]. Here we report the first application of SAT techniques to metabolic problems.

The SPABBATS algorithm was applied here to a specific problem, the analysis of glutamate metabolism in *B. subtilis*. However, the solution strategies are applicable to a broad spectrum of metabolic problems. For instance, SPABBATS can be particularly useful in the characterization of suppressor mutants. Moreover, SPABBATS can also be useful in synthetic biology. Although used here to find pathways in a reconstruction of the metabolism of *B. subtilis*, it is also possible to use a database of enzymes as the starting model. In this way, it can be used to construct synthetic pathways that satisfy specific input-output and mass-balance requirements.

## Methods

### Algorithm for finding short pathways between a basis and a target set of metabolites (SPABBATS)

Our approach draws inspiration from flux-balance analysis (FBA [21]) in the sense that it searches the flux space of a metabolic network for fluxes that comply with a set of stoichiometric constraints. The major difference to FBA lies in the optimality criterion; in FBA the value to optimize is the target flux. In our case we change from optimization to satisfiability: we search for a flux that satisfies all the constraints, including a maximum number of allowed reactions.

Another important difference, that is a consequence of satisfiability approach, is that we use two variables for each flux instead of one. The first variable is a positive integer, which is a relative measure of the contribution of that particular flux to the total pathway. The second variable is Boolean and defines whether or not the particular flux takes part in the solution.

As in FBA, we define **S **as the stoichiometric matrix of the network with *n *reactions and *m *compounds. Reversible reactions are split into two unidirectional reactions. We divide the set of compounds into three disjoint sets:

i) **B **is the set of basis compounds that are considered freely available, either because they are provided in the medium, or because they are "currency metabolites", whose concentration is buffered by the whole system (e.g. ATP, ADP, NADH, etc.)

ii) **T **is the set of target compounds, the ones constrained to be produced in the pathways of interest

iii) **I **is the set containing all other compounds, that can be intermediates of the resulting pathway

We use different constraints for each of these sets. The compounds in the set **B **are left unconstrained. For each compound in the set **T**, we write a constraint in the form:

(1) $$\sum_{i=1}^{n}s_{ij}a_{i}b_{i}>0,$$

where *s*_ij _is the stoichiometric coefficient of compound *j *in reaction *i*, and *a*_i _and *b*_i _are the integer and Boolean valued variables of reaction *i*, respectively. These constraints mean that in the solution pathway the overall flux to these metabolites should be positive.

For the compounds in the set **I **we use a constraint similar to (1), with the difference that we use a "greater than or equal to" (≥) sign. In FBA, an equality sign is used here, to constraint the fluxes to the steady-state space. We purposely do not constrain the pathway to the steady-state space, since the candidate solutions to the problem will not be the only pathway active in the cell and the intermediates that are accumulated in our pathway can be used by other pathways operating in parallel in the system. We require the total flux to these compounds to be non-negative, since the supposition is that they are not present in sufficiently high amounts to allow sustained growth on their consumption.

Next, we add constraints that limit the directionality of reversible reactions. This is done with constraints in the form:

(2) $$b_{i}+b_{j}<2,$$

where *b*_i _and *b*_j _are the Boolean variables of two reactions that together characterize a reversible reaction. These constraints mean that no two directions of a reversible reaction can appear in the final pathway at the same time.

Last, we add a constraint for the total length of the solution. This constraint is:

(3) $$\sum_{i=1}^{n}b_{i}\leq k,$$

where *k *is a positive integer value that determines the maximum number of reactions that can appear in the pathway. This constraint does not immediately find the best solution, but it puts successively stricter upper-bounds to the maximum number of reactions that are allowed. Thus, it is able to find the shortest solution after some iterations by choosing successively smaller numbers for *k*.

The constraints for the compounds in **T **and **I **are not linear, since each term in the sum is composed of two variables instead of one. For this reason, a linear optimization strategy cannot be used directly. This limitation is not present when we use the SAT-solver HySAT [30]. It is able to find assignments to the variables that satisfy all the constraints in the system, even when these are non-linear. It is also able to detect if no such assignment exists.

If the shortest solution has been found, the best sub-optimal solution can be found by adding an additional constraint in the form:

(4) $$\sum_{i\in K}b_{i}<k_{op},$$

where *k*_op _is the number of reactions in the shortest solution and *K *is the set of indices for the reactions in the shortest solution. In other words, we constrain the sum of all the Boolean variables of the optimal solution to be less than *k*_op_, thus leaving out the shortest solution from the solution space. By iterating this process with the Boolean variables of the sub-optimal pathway, we can find solutions with successively higher number of reactions.

The particular implementation of this algorithm for the problem mentioned in the Results section is as follows: we used the genome-scale reconstruction of *B. subtilis *[9]. We removed the biomass "reaction"; it is useful for FBA, since it describes the target flux to cellular growth, but is meaningless in our context. In addition, we removed the reaction "glutamate dehydrogenase" (R_GLUDxi) to simulate the conditions of the strain GP717. We also scaled the non-integer stoichiometric coefficients of the model to integer values (and divided by the greatest common denominator). In our case, the set **B **contained the metabolites ATP, ADP, NAD^+^, NADH, FAD, FADH_2_, H_2_0, H^+^, NH_4_^+^, and glutamate. These "currency metabolites" were chosen due to their participation in most catabolic pathways in the cell. The set **T **contained just 2-oxoglutarate. The remaining compounds were assigned to the set **I**. We set the interval for the *a*_*i *_to [[1], 1000]. The calculations were done using an Intel Core2 Duo processor at 2.66 GHz, with 3.25 GB of RAM. The first pathway (the one involving leucine as intermediate) was found after 28 seconds. All other pathways took less than 8 minutes each to calculate.

### Bacterial strains and growth conditions

All *B. subtilis *strains used in this work are derived from the laboratory wild type strain 168. They are listed in Table 1. *E. coli *DH5α [31] was used for cloning experiments. *B. subtilis *was grown in C minimal medium containing ammonium as basic source of nitrogen [32]. Glutamate and/or glucose were added as carbon source as indicated. The medium was supplemented with auxotrophic requirements (at 50 mg/l). *E. coli *was grown in LB medium and transformants were selected on plates containing ampicillin (100 μg/ml). LB, SP and CSE plates were prepared by the addition of 17 g Bacto agar/l (Difco) to LB, SP or CSE medium, respectively.

**Table 1:** *B. subtilis *strains used in this study

Strain	Genotype	Source or Reference
GP28	*trpC2 *Δ*gudB*::*cat rocG*::Tn*10 spc amyE*::(*gltA-lacZ aphA3*)	[14]
GP717	*trpC2 *Δ*gudB*::*cat rocG*::Tn*10 spc amyE*::(*gltA-lacZ aphA3*) *gltB1 ansR*-C107A	[11]
GP811	*trpC2 *Δ*gudB*::*cat rocG*::Tn*10 spc amyE*::(*gltA-lacZ aphA3*) Δ*ansR*::*tet*	see Materials and methods
GP1154	*trpC2 *Δ*gudB*::*cat rocG*::Tn*10 spc amyE*::(*gltA-lacZ aphA3*) *gltB1 ansR*-C107A Δ*ansAB*::*ermC*	see Materials and methods

### DNA manipulation and transformation

Transformation of *E. coli *and plasmid DNA extraction were performed using standard procedures [31]. Restriction enzymes, T4 DNA ligase and DNA polymerases were used as recommended by the manufacturers. DNA fragments were purified from agarose gels using the Nucleospin Extract kit (Macherey and Nagel, Germany). Phusion DNA polymerase was used for the polymerase chain reaction as recommended by the manufacturer. All primer sequences are provided as supplementary material (Additional File 1 Table S1). DNA sequences were determined using the dideoxy chain termination method [31]. All plasmid inserts derived from PCR products were verified by DNA sequencing. Chromosomal DNA of *B. subtilis *was isolated as described [33].

*E. coli *transformants were selected on LB plates containing ampicillin (100 μg/ml). *B. subtilis *was transformed with plasmid DNA or PCR products according to the two-step protocol described previously [33]. Transformants were selected on SP plates containing tetracyclin (Tet 10 μg/ml), or erythromycin plus lincomycin (Em 2 μg/ml and Lin 25 μg/ml).

### Plasmid and mutant strain construction

To express a plasmid-borne *ansR *gene in *B. subtilis*, we constructed plasmid pGP873. For this purpose the *ansR *gene was amplified with the primers KG18 and KG19 using chromosomal DNA of *B. subtilis *as a template. The PCR product was digested with *Bam*HI and *Sal*I and cloned into the overexpression vector pBQ200 [34].

Deletion of the *ansAB *and *ansR *genes was achieved by transformation with PCR products constructed using oligonucleotides to amplify DNA fragments flanking the target genes and an intervening erythromycin and tetracyclin resistance cassettes from plasmids pDG647 and pDG1514, respectively, [35] as described previously [36]. The PCR products were used to transform GP717 and GP28 for the deletion of the *ansAB *and *ansR*, respectively.

### Reverse transcription-real-time quantitative PCR

For RNA isolation, the cells were grown to an OD_600 _of 0.5 - 0.8 and harvested. Preparation of total RNA was carried out as described previously [37]. cDNAs were synthesized using the One-Step RT-PCR kit (BioRad) as described [38]. Real time quantitative PCR was carried out on the iCycler instrument (BioRad) following the manufacturer's recommended protocol by using the primers KG26/KG27 for the *ansA *gene, KG38/KG39 for the *ald *gene and KG40/KG41 for the *bcd *gene, respectively. Their recommended data analysis procedure was also used. The *rpsE *and *rpsJ *genes encoding constitutively expressed ribosomal proteins were used as internal controls and were amplified with the primers *rpsE*-RT-*fwd/rpsE*-RT-*rev *and *rpsJ*-RT-*fwd/rpsJ*-RT-*rev*, respectively. The expression ratios were calculated as fold changes as described [38]. RT-PCR experiments were performed in duplicate.

## Authors' contributions

LAF, KG, and JS planned the project. LAF and RP conceived and implemented the SPABBATS algorithm. KG and ST provided the experimental validation of the method. LAF, KG, and JS wrote the manuscript and created the Table and Figures. All authors read and approved the final manuscript.

## Supplementary Material

Additional file 1 **Supplementary Table S1**. This Table lists the sequences of the oligonucleotide primers used in the experiments. Click here for file
