# Supplementary material for: SPABBATS: A pathway-discovery method based on Boolean satisfiability that facilitates the characterization of suppressor mutants
Source: BMC Syst Biol. 2011 Jan 11;5:5. doi: 10.1186/1752-0509-5-5 (PMC3024933; doi:10.1186/1752-0509-5-5)
Supplement: Additional file 1 — Supplementary Table S1. This Table lists the sequences of the oligonucleotide primers used in the experiments. [file 1752-0509-5-5-S1.PDF]

SPABBATS: A pathway-discovery method based on Boolean satisfiability that facilitates the characterization of suppressor mutants

Lope A. Flórez, Katrin Gunka, Rafael Polanía, Stefan Tholen, and Jörg Stülke

Table S1: Primer sequences used in the experiments

Name	Sequence
KG12	5’ CCTATCACCTCAAATGGTTCGCTGGATGGCCAGCCGCTGAGTGAAG
KG13	5’ CCGAGCGCCTACGAGGAATTTGTATCGCCGAGAAGGTCAGCTGTATA TTGAAGC
KG14	5’ ACCTCGTAAATGCTCATGTCTTCGCC
KG15	5’ CCGGAAGTCATTCTAGAGCTTGAGGA
KG18	5’AAAGGATCCCAGCTCAAGGTGAAAAAGGAGCGGAA
KG19	5’TTTGTCGACTCATTAACTCAGTTCCTCCTGTACTTTTCTTTTTGTG
KG25	5’ TTGAAGGGGAAAATGGGCTG
KG26	5’ CTATTTCCACCCAGTATTCAGG
KG28	5’ ATGGCTTGGACCCGTTATTGGGG
KG29	5’ CCTATCACCTCAAATGGTTCGCTGGAGCCAGCCCATTTTCCCCTTC
KG30	5’ CCGAGCGCCTACGAGGAATTTGTATCGCGGCGCTGATCATCTTGTT GATG
KG31	5’ AAGTCGGCACAACGCCTCCGG
KG38	5’ CCGTGTCGCATTAACACC
KG39	5’ ACCTGCTTCGGATCAGCA
KG40	5’ TAAACCTTGGCGGCGGAA
KG41	5‘ CCATATCCTCGACCGTTG
rpsJ-RT-fwd	5` GAAACGGCAAAACGTTCTGG
rpsJ-RT-rev	5` GTGTTGGGTTCACAATGTCG
rpsE-RT-fwd	5`GCGTCGTATTGACCCAAGC
rpsE-RT-rev	5` TACCAGTACCGAATCCTACG
mls-fwd (kan)	5`CAGCGAACCATTTGAGGTGATAGGGATCCTTTAACTCTGGCAACCCTC
mls-rev (kan)	5`CGATACAAATTCCTCGTAGGCGCTCGGGCCGACTGCGCAAAAGACAT AATCG
Tc fwd1 (kan)	5`CAGCGAACCATTTGAGGTGATAGGGCTTATCAACGTAGTAAGCGTGG
Tc rev (kan)	5`CGATACAAATTCCTCGTAGGCGCTCGGGAACTCTCTCCCAAAGTTGAT CCC
